# Public practices on antibiotic use: A cross-sectional study among Qatar University students and their family members

**DOI:** 10.1371/journal.pone.0225499

**Published:** 2019-11-26

**Authors:** Ghadir Fakhri Aljayyousi, Manar E. Abdel-Rahman, Asmaa El- Heneidy, Rana Kurdi, Eman Faisal

**Affiliations:** 1 Department of Public Health, College of Health Sciences, Qatar University, Doha, Qatar; 2 School of Medicine, Griffith University, Gold Coast, Australia; 3 Graduate Assistant at the Department of Public Health, College of Health Sciences, Qatar University, Doha, Qatar; University of Campania, ITALY

## Abstract

**Background:**

Antimicrobial resistance (AMR) is a serious public health problem and a global concern. The inappropriate use of antibiotics has been identified by the World Health Organization as a major risk factor for AMR.

**Methods:**

The purpose of this research study is to assess the prevalence of inappropriate antibiotic use among Qatar University students and their family members, detect sociodemographic factors associated with inappropriate use, evaluate the knowledge and attitude towards antibiotic use, and assess respondents’ opinions on healthcare providers’ antibiotic prescription practices. Participants (N = 596) completed a self-administered questionnaire. Descriptive analysis, the Pearson chi-squared test, and multivariate logistic regression analyses were performed.

**Results:**

The major inappropriate antibiotic use practices followed by the respondents were using antibiotics without prescription (82%), not completing the antibiotic course (45%), and obtaining antibiotics from the pharmacy without prescription (23%). The chi-square test results showed that age (p = 0.031) and nationality (p = 0.041) were associated with using antibiotics without prescription. In addition, respondents less than 21 years of age (p<0.001), who had only a secondary education (p = 0.007), and who lived in one of the large and crowded cities in Qatar (p = 0.011) had higher odds of stopping the antibiotic before completing the course. Our study also revealed that almost 60% of the respondents had inadequate knowledge and a negative attitude towards antibiotic use. Nationality and municipality were the independent factors associated with having appropriate knowledge of antibiotic use. Univariate logistic regression analyses in our study demonstrated that older (>26 years), married and university-graduated participants were more likely to have a positive attitude towards antibiotic use than others. Respondents also reported that neither doctors nor pharmacists were providing adequate patient education about appropriate antibiotic use. The Socio-Ecological Model was applied to interpret the findings and frame implications.

**Conclusion:**

The findings shed light on various factors shaping antibiotic use practices and provide evidence to design multilevel behavioral interventions to improve public practices of antibiotic use.

## Introduction

Antibiotics are drugs used to stop and manage bacterial infections, either by killing the infectious bacteria inside the host body or by preventing their multiplication and spread, allowing the host’s own defense to contain and eliminate it [1]. Antibiotic resistance (ABR) is the ability of bacteria to resist the effects of antibiotics and is one of the biggest threats to human health [2]. Infections with resistant organisms are difficult to treat, requiring costly and sometimes poisonous alternatives [3]. The main reasons for the development of bacterial resistance are the overuse and the misuse of antibiotics. Examples are the use of antibiotics when they are not really needed, the discontinuation of the antibiotic course of treatment before completion and the use of antibiotics in improper doses [4].

According to the World Health Organization’s new global antimicrobial surveillance system, ABR is widely spread, existing among 500,000 people in 22 countries; it kills thousands of individuals every year [5]. In the US, approximately two million cases of ABR infections and at least 23,000 deaths are reported annually, according to the Centers for Disease Control and Prevention’s (CDC) report in 2013 [5]. A European study estimated that more than 33,000 people in Europe die from ABR infections each year [6]. In developing countries, the level of drug-resistant infections and deaths are almost the same as or higher than in developed countries [2]. Patients with these infections will stay longer in the hospital and suffer from more side effects, thus contributing to higher health care costs. The CDC estimated the economic impact of ABR in the US alone to be approximately US $35 billion per year [5].

Few studies have reported on the emergence of ABR in the Gulf Cooperation Council (GCC) countries [7, 8]. During 1999–2003, Kuwait had the fastest growth rate of ABR after China and the USA [9]. This rise in ABR was accredited mainly to inappropriate prescribing of antibiotics and overuse of antibiotics in self-medication [9]. In Saudi Arabia, in-depth interviews were carried out to understand consumers’ perspectives on nonprescribed antibiotic use, which showed that self-medication with antibiotics was associated with ABR, adverse events and treatment failure [10]. This was due to lack of knowledge about ABR, consumer health beliefs and practices and difficulty accessing healthcare facilities [10].

An increase in consumption, longer duration and multiple courses of antibiotics have been associated with the development of resistance [11, 12]. Each time an antibiotic is prescribed and used inappropriately, ABR will accelerate. Appropriate prescription of antibiotics refers to “prescribing the right group of antibiotics for the given diagnosis, at the right dose, at the right time, and for the right duration” [13]. Inappropriate antibiotic use in the healthcare system is severe in developed countries [14–16]. In the US, approximately half of the patients who have acute respiratory infections not requiring antibiotics leave the clinic with an antibiotic prescription [14]. Approximately 11.4 million unnecessary antibiotic prescriptions, which account for 29% of total prescriptions, are prescribed for children with acute respiratory tract infections annually [17]. A study in China reported that two-thirds of hospitalized patients received antibiotics in their treatment [18]. In some countries, such as India and Kenya, children who suffer from diarrhea are treated with oral rehydration and are also given antibiotics [19]. Al- Niemat and her colleagues reported that 35% of prescribed medications from an outpatient clinic in Jordan were antibiotics [20].

Self-medication/prescription of antibiotics is another issue associated with the risk of inappropriate drug use. In the Middle East region, a recent systematic review showed that the prevalence of self-medication and self-prescription with antibiotics ranges from 19% to 82% across studies [21]. Penicillin, usually used to treat upper respiratory tract problems, is the most commonly used antibiotic. It was mainly obtained without prescriptions from pharmacies, leftover drugs and relatives or friends. In addition, inappropriate drug use was reported, including sharing of antibiotics and storing of antibiotics at home for later use [21].

At the community level, previous research found extensive problems in knowledge, attitudes, beliefs and behaviors that influence antibiotic use [22–24]. Patients and community members may take unnecessary antibiotics, not finish the full course, share medications with other people, and/or keep part of the course for a later illness [25]. In some countries, such as Nigeria, Sudan and Bangladesh, nearly all antibiotics consumed are apparently taken without a prescription. Even some developed countries, such as Italy, Spain and Greece, have levels of over-the-counter use that reach 20% of the sold antibiotics [26].

A systematic review conducted in Qatar to explore antibiotic use in upper respiratory tract infections for patients visiting healthcare settings revealed overprescribing of antibiotics in all health care settings [27]. For instance, a high prevalence of antibiotic misuse for respiratory tract infections was recorded in the private sector (85%). Similarly, in the medical intensive care unit, antibiotics were 76% of prescribed drugs, in which respiratory tract infections accounted for 57% of prescriptions [27]. Moreover, a cross-sectional study was conducted in Qatar to assess the perceptions and practices of physicians and pharmacists regarding antibiotic misuse at primary healthcare centers. The study asserted that 90.7% of physicians and 87.8% of pharmacists perceived antibiotic misuse as a major public health issue. Furthermore, the majority of physicians and pharmacists believed in patient education as a way to prevent antibiotic misuse [28]. A recent cross-sectional study targeted participants at two community pharmacies to evaluate their knowledge and views towards antibiotic use in Qatar and reported misconceptions and inappropriate use of antibiotics among participants [29].

In Qatar, a country with highly mobile inhabitants, one of the reasons for inappropriate use of antibiotics is that residents can purchase antibiotics without prescription when traveling and bring them back to Qatar [2]. A Qatari qualitative study of pharmacists’ perception of antibiotic use reported the misconceptions and inappropriate practices of antibiotic use among the public. The majority of the participants in the study declared that patients had a desire to use antibiotics when not indicated or prescribed to treat viral and other self-limiting diseases [30]. Noncompliance with prescribed directions was also reported. This included discontinuation of antibiotics before they were finished based on self-evaluation of symptoms and using higher doses of antibiotics to get better faster [30].

Inappropriate antibiotic use is driven by individuals’ behaviors and practices. A better understanding of these practices will contribute to achieving a positive change for the benefit of patients and community members’ health. There is a paucity of studies regarding antibiotic use practices among community members in Qatar and the factors influencing these practices. Our study will be the first of its kind in assessing antibiotic use practices among college students in Qatar. This study will provide baseline data for future studies and provide insight into the development of behavioral interventions with the aim of optimizing appropriate antibiotic use among the public, which will, in turn, help in the implementation of the antibiotic stewardship program in Qatar and thus decrease the rate of ABR.

The purpose of this study is to assess public practices on antibiotic use among specific communities represented by Qatar University students and their family members. More specifically, the study objectives are to measure the degree of antibiotic prescribing; assess knowledge, attitudes and practices on antibiotic use; evaluate participants’ opinions on healthcare provider antibiotic prescription practices; and quantify how all these factors are associated with the study population characteristics.

## Materials and methods

### Study design, sampling and sample size

This is a cross-sectional study that was conducted between September 2018 and March 2019. Using a convenience-sampling design, three colleges out of nine in the university were chosen to recruit participants: the College of Engineering, the College of Arts and Sciences, and the College of Health Sciences. Next, students expected to have diverse fields of study and nationalities were recruited from two main female buildings as well as from the female students’ dormitory at the university. Participants were eligible to be included in the study if they were ≥18 years old, were aware of the term “antibiotic” and understood English or Arabic. Additionally, in order to enhance external validity, one family member of each selected student was asked to complete the questionnaire. The family member was also chosen using a convenience-sampling method. The study was approved by the Qatar University Institutional Review Board (Research Ethics Approval No. QU-IRB715-E/16).

Participants and their family members completed a total of 596 questionnaires. Using an anticipated prevalence of 73% of antibiotic self-medication [31] and a 4% margin of error and allowing for a 20% nonresponse rate, the aim was to collect data from 600 (rounded from 592) participants [32]. The majority of the study participants were university students (64.4%). Approximately 86% of the students were females, and more than 60% of all the participants were aged 21 years and above. The majority were single (74%), undergraduate and unemployed students who resided in Doha. Our sample can not be considered representative of the larger community at the university.

### Data collection

Six senior public health students from the College of Health Sciences (CHS) at Qatar University were the main data collectors, who invited other students to participate in the study. Participants who agreed to participate in the study were provided an anonymous self-administered questionnaire to be completed by themselves and by one of their family members. Data collectors assured participants of confidentiality and provided them with written consent to participate in the study. No incentives were offered for participation.

The questionnaire was adapted from the ABR: Multi-Country Public Awareness Survey [33] and a validated questionnaire from a previous study in the region [34]. It was modified to suit the study objectives and the local population to assure its applicability. One member of the study team translated the questionnaire into Arabic. To ensure the accuracy of the translation, a different member back-translated it into English. To determine its effectiveness, the questionnaire was pretested on a group of 20 students not included in the study. A group of two experts from CHS who were familiar with the questionnaire contents and design were involved in this process.

The questionnaire included four sections: demographics; antibiotic prescription history and antibiotic self-medication; knowledge and attitude towards antibiotic use; and opinion on healthcare providers’ antibiotic prescription practices.

The first section of the questionnaire consisted of the sociodemographic questions. The first two questions identified the participant as a student or a family member and their relationship to the student. There were questions about gender, age, marital status, nationality, highest educational level completed, employability status, area of residence, and total household income. These questions were adapted from the Kuwaiti study [34] and WHO survey [33].

The second section covered antibiotic prescriptions, antibiotic use practices and self-medication with 14 close-ended questions. The first eight questions asked: if they had been prescribed antibiotics within the last year; the frequency of being prescribed antibiotics during the last year; if they had finished the last antibiotic prescribed; if not, they were asked about the reasons; whether they had used an antibiotic without being prescribed it by a doctor or dentist within the last year (self-medication); frequency of self-medication with an antibiotic during the last year; conditions for which the antibiotics were self-prescribed; and if they completed these self-prescribed antibiotics. Additionally, a question listed seven conditions for which antibiotics were used without prescription, and respondents were asked to check all the conditions for which they had self-medicated with antibiotics. Other questions asked if the participant used antibiotics that were originally prescribed for an infection that recurred later, originally prescribed for another type of infection, obtained from a pharmacy abroad without a prescription, obtained from a pharmacy within Qatar without a prescription, originally prescribed for another family member, or prescribed for someone else who was not a family member. These questions were mainly adapted from the Kuwaiti study. The questions about antibiotic prescription, the conditions for which antibiotics were used, and where participants usually obtained antibiotics were also included in the WHO survey.

In the third section, there were 16 statements about knowledge and attitudes regarding antibiotic use. A 5-point Likert scale, which started from strongly disagree to strongly agree, was used to assess the participants’ responses. The first nine statements assessed knowledge about antibiotic use among the participants. Participants were asked if they agreed with these statements or not: Different antibiotics are needed to cure different diseases; antibiotics are effective against bacteria; Antibiotics can kill the bacteria that normally live on the skin and in the gut; Antibiotics speed up the recovery from most coughs and colds; antibiotics work on most coughs and colds; Antibiotics are effective against viruses; If they get side effects during a course of antibiotic treatment, they should stop taking them as soon as possible; If they get some kind of skin reaction when using an antibiotic, they should not use the same antibiotic again; and Antibiotic use can cause imbalance in the body’s own bacterial flora. These statements were adapted from the Kuwaiti study.

The remaining seven statements in this section assessed the participants’ attitudes towards antibiotic use. Participants were asked if they agreed with these statements or not: I always complete the course of treatment with antibiotics even if I feel better; It is good to be able to get antibiotics from relatives or friends without having to see a medical doctor; I prefer to be able to buy antibiotics from the pharmacy without a prescription; I prefer to keep antibiotics at home in case there may be a need for them later; If I feel better after a few days, I sometimes stop taking my antibiotics before completing the course of treatment; I prefer to use an antibiotic if I have a cough for more than a week; and If I have a sore throat, I prefer to use an antibiotic. These questions were mainly adapted from the Kuwaiti study. The two questions on attitudes about obtaining antibiotics from relatives and friends and stopping antibiotics before completing the course of treatment were also included in the WHO survey.

Section four included four statements to assess participants’ opinions regarding healthcare providers’ antibiotic prescription practices. They were asked if they agreed with these statements or not: Pharmacists often tell you how antibiotics should be used; Doctors often take time to inform the patient during the consultation how antibiotics should be used; Doctors often prescribe antibiotics because the patient expects it; and Doctors often take time to consider carefully whether antibiotics are needed or not. A 5-point Likert scale, which started from strongly disagree to strongly agree, was used to assess the participants’ responses. All questions were adapted from the Kuwaiti study, and the question on the role of doctors and pharmacists in explaining how a patient should use an antibiotic was also included in the WHO survey.

### Study variables

The main objectives of the study were to measure the degree of antibiotic prescribing, assess inappropriate antibiotic use practices, assess knowledge and attitudes on antibiotic use, evaluate respondents’ opinions on healthcare providers’ antibiotic prescription practices, and evaluate how all these factors are associated with the respondents’ characteristics.

The main outcome variable to measure the degree of antibiotic prescribing was being prescribed antibiotics 1 or more times within the last year (Yes, No). Inappropriate antibiotic use and self-medication were measured using three variables: Using an antibiotic without prescription (Yes, No), not completing the last prescribed or unprescribed antibiotic course (Yes, No) and obtaining an antibiotic without a prescription from a pharmacy abroad or in Qatar (Yes, No).

Two outcome variables, capturing knowledge and attitude towards antibiotic use, were derived using a scoring system. The system assigned scores of 1 or zero to each participant response to the relevant statements. A continuous total score was computed for each respondent as the sum of their 0/1 scores. This total score was further categorized into a binary variable using the median of the total score as a cutoff point.

Knowledge on antibiotic use was based on responses to the nine knowledge statements asked. A participant who responded to a correct statement with “strongly agree” or “agree” was given a 1 score and zero otherwise. For wrong statements, a score of 1 was given to participants who responded “strongly disagree” or “disagree” and zero otherwise. The total score (range 0–9) was categorized as acceptable knowledge for total scores equal to or greater than the median and unacceptable knowledge otherwise.

The attitude towards antibiotic use was derived from participants’ responses to the seven attitude statements. Positive statements with “strongly agree” or “agree” were given a 1 score and zero otherwise, while negative statements were given a score of 1 to participants who responded “strongly disagree” or “disagree” and zero otherwise. The total score (range 0–7) was categorized as a positive attitude for total scores equal to or greater than the median and a negative attitude otherwise.

Furthermore, opinions on healthcare provider antibiotic prescription practices was captured using six opinion statements. Additionally, the basic characteristics of the participants, such as age, gender, nationality, marital status, highest educational level completed, employability status, area of residence, and total household income (Table 1), were considered as independent variables.

**Table 1:** Sociodemographic characteristics of respondents (n = 596).

Characteristic	Frequency	Percentage (%)
Participant type		
Student	384	64.4
Family member	206	34.6
Missing	6	1.0
Sex		
Male	69	11.6
Female	514	86.2
Missing	13	2.2
Age in years		
<21	228	38.3
21–25	230	38.6
26+	138	23.2
Marital status		
Single	440	73.8
Married	125	21.0
Missing	31	5.2
Nationality		
Qatari	241	40.4
Other GCC	46	7.7
Other Arab	243	40.8
Non-Arab	22	3.7
Missing	44	7.4
Highest level of education		
Secondary	318	53.4
University+	278	46.6
Employment		
Yes	143	24.0
No	435	73.0
Missing	18	3.0
Municipality		
Doha	284	47.7
Al-Rayan	163	27.3
Elsewhere	117	19.6
Missing	32	5.4
Household income		
1	90	15.1
2	165	27.7
3	109	18.3
4	184	30.9
6	1	0.2
Missing	47	7.9

### Statistical analysis

After data collection, data were checked in the paper questionnaires for potential mistakes and inconsistencies. Each questionnaire was given a unique identification. Data were then entered into the *epidata* entry program*. To ensure data entry quality, the data entry program was designed to perform range calculations, consistency checks, field validations and data-type restrictions. Data were then exported into the Stata data format for analysis.

Entered data were cleaned and analyzed using the Statistical Package *Stata* version 15. Means and standard deviations were used to summarize continuous data, while proportions and percentages were used for categorical variables. Association of respondents’ characteristics with antibiotic prescribing, inappropriate antibiotic use and self-medication, and knowledge and attitudes towards antibiotic use were evaluated using univariate logistic regression. All variables with a global Wald test p < 0.25 in the univariate analysis were included in an initial multivariable logistic regression analysis to determine the factors that were independently associated with each outcome variable. Based on this model, a final model was built, considering a global test p-value of 0.05 to be statistically significant.

## Results

### Sociodemographic characteristics of the respondents

A total of 600 subjects were approached to participate in the study, and 596 of them completed the questionnaire. The majority of the respondents were females (86.2%). Approximately two-thirds of the respondents were students (64.4%), and 23.2% were aged 26 years or above. Among these respondents, 73.8% were single, and only 3.7% were non-Arab. Key characteristics of the respondents are presented in Table 1.

### Antibiotic use

**Fig 1:**
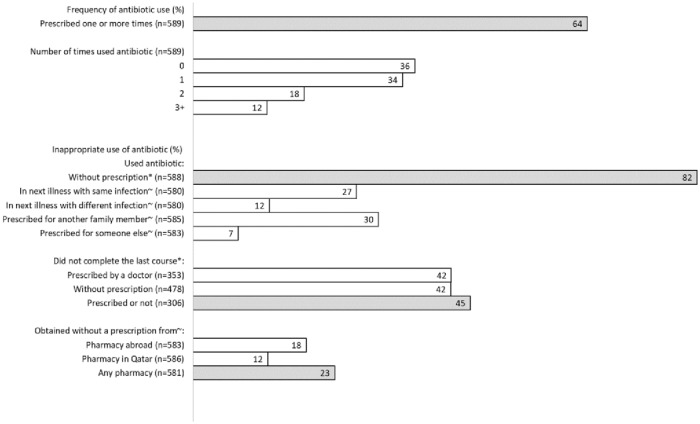
Degree of antibiotic prescribing and inappropriate antibiotic use.

Of all respondents, 64% had received at least one prescription for antibiotics within the last year prior to our study, 34% of whom had used antibiotics once, and 12% had used them three times or more. During the same period, the majority of the respondents (82%) used antibiotics without prescription, 37% of whom used antibiotics that were prescribed for another family member or someone else and 27% of whom used antibiotics that were prescribed for them for a previous illness when they had similar symptoms later on. In addition, 45% of the respondents did not complete the last course of antibiotics, and 23% obtained the antibiotic without prescription from any pharmacy (Fig 1).

Slight significant differences in using antibiotics with prescription among the respondents in relation to age were reported (p = 0.048). Respondents with older age (>26 years old) had higher odds ratios of using antibiotics with prescriptions than youngsters (Table 2). However, none of the variables was significantly associated with using antibiotics with prescriptions at the 0.05 level of significance.

**Table 2:** Association between respondents’ characteristics and using antibiotics with prescriptions.

	Total N = 589	Was prescribed antibiotic^^^ N = 375	p-value^^^^	OR [95% CI] ^^^^^
Participant type*			0.510	
Student	381	238 (62.5%)		1.00
Family member	204	133 (65.2%)		1.13 [0.79,1.61]
Sex*			0.320	
Male	69	48 (69.6%)		1.00
Female	508	322 (63.4%)		0.76 [0.44,1.30]
Age in years			0.048	
<21	226	139 (61.5%)		1.00
21–25	225	136 (60.4%)		0.96 [0.66,1.40]
26+	138	100 (72.5%)		1.65 [1.04,2.61]
Marital status*			0.340	
Single	434	270 (62.2%)		1.00
Married	124	83 (66.9%)		1.23 [0.81,1.87]
Nationality**			0.940	
Qatari	238	155 (65.1%)		1.00
Other GCC	46	31 (67.4%)		1.11 [0.57,2.17]
Other Arab	240	152 (63.3%)		0.92 [0.64,1.34]
Non-Arab	21	13 (61.9%)		0.87 [0.35,2.18]
Highest level of education			0.250	
Secondary	312	192 (61.5%)		1.00
University+	277	183 (66.1%)		1.22 [0.87,1.71]
Employment*			0.120	
Yes	143	99 (69.2%)		1.00
No	432	268 (62.0%)		0.73 [0.48,1.09]
Municipality*			0.370	
Doha	281	185 (65.8%)		1.00
Al-Rayan	163	98 (60.1%)		0.78 [0.53,1.17]
Elsewhere	116	78 (67.2%)		1.07 [0.67,1.69]
Household income**			0.560	
1	89	52 (58.4%)		1.00
2	163	104 (63.8%)		1.25 [0.74,2.13]
3	109	74 (67.9%)		1.50 [0.84,2.69]
4	182	119 (65.4%)		1.34 [0.80,2.26]

^^^Data are presented as n (row %),

^^^^Chi-square test,

^**^^^**^Crude logistic regression,

*0.7–5.5% missing,

**8.1–8.5% missing

The chi-square test results showed that nationality (p = 0.041) and age (p = 0.031) were associated with using antibiotics without prescription among the respondents (Table 3). However, using multivariate logistic regression revealed that only nationality was a statistically significant predictor of antibiotic use without prescription (p = 0.033): non-Arab respondents had lower odds of consuming antibiotics without prescriptions than Qataris (OR = 0.29, 95% CI: 0.11, 0.75).

**Table 3:** Association between respondents’ characteristics and using antibiotics without prescriptions.

	Total N = 588	Used antibiotic without prescription^^^ N = 481	p-value^^^^	OR [95% CI]^^^^^
Participant type*			0.130	
Student	379	316 (83.4%)		1.00
Family member	203	159 (78.3%)		0.72 [0.47,1.11]
Sex*			0.910	
Male	67	55 (82.1%)		1.00
Female	508	414 (81.5%)		0.96 [0.49,1.87]
Age in years			0.031	
<21	225	196 (87.1%)		1.00
21–25	226	178 (78.8%)		0.55 [0.33,0.91]
26+	137	107 (78.1%)		0.53 [0.30,0.93]
Marital status*			0.170	
Single	434	362 (83.4%)		1.00
Married	123	96 (78.0%)		0.71 [0.43,1.16]
Nationality**			0.041	
Qatari	237	201 (84.8%)		1.00
Other GCC	46	40 (87.0%)		1.19 [0.47,3.02]
Other Arab	240	193 (80.4%)		0.74 [0.46,1.18]
Non-Arab	21	13 (61.9%)		0.29 [0.11,0.75]
Highest level of education			0.063	
Secondary	317	268 (84.5%)		1.00
University+	271	213 (78.6%)		0.67 [0.44,1.02]
Employment*			0.190	
Yes	141	110 (78.0%)		1.00
No	429	356 (83.0%)		1.37 [0.86,2.20]
Municipality*			0.960	
Doha	281	229 (81.5%)		1.00
Al-Rayan	160	131 (81.9%)		1.03 [0.62,1.70]
Elsewhere	116	96 (82.8%)		1.09 [0.62,1.92]
Household income**			0.056	
1	89	75 (84.3%)		1.00
2	163	129 (79.1%)		0.71 [0.36,1.40]
3	108	81 (75.0%)		0.56 [0.27,1.15]
4	182	158 (86.8%)		1.23 [0.60,2.51]

^^^Data are presented as n (row %),

^^^^Chi-square test,

^**^^^**^Crude logistic regression,

*1.0–5.6% missing,

**8.1–8.5% missing

Concerning adherence to the recommended antibiotic regimen, forty-five percent of the study population reported that they did not complete their last course of antibiotics (Fig 1). Table 4 presents crude analysis showing associations between not completing the last antibiotic course and study variables. In multivariable logistic regression analysis (table not shown), age (p<0.001), level of education (p = 0.007) and municipality (p = 0.011) were significant predictors of adherence to the antibiotic course. Compared to the youngest age group (<21 years old), the other two age groups had lower odds of stopping the antibiotic course early (OR = 0.41, 95% CI: 0.23–0.73; and OR = 0.25, 95% CI: 0.13–0.49) for the 21-25-year and ≥26-year groups, respectively. Respondents who had a secondary education had lower odds of completing their antibiotic courses than those with university degrees (OR = 0.50, 95% CI: 0.30–0.83). Additionally, compared to the respondents who lived in Doha, those who lived in Al-Rayan had 2.4 times higher odds of stopping their antibiotic course early (OR = 2.4, 95% CI: 1.32–4.40).

**Table 4:** Association between respondents’ characteristics and not completing the antibiotic course.

	Total N = 306	Did not complete last antibiotic course^^^ N = 139	p-value^^^^	OR [95% CI] ^^^^^
Participant type*			0.150	
Student	191	92 (48.2%)		1.00
Family member	111	44 (39.6%)		0.71 [0.44,1.14]
Sex*			0.830	
Male	39	17 (43.6%)		1.00
Female	264	120 (45.5%)		1.08 [0.55,2.12]
Age in years			<0.001	
<21	109	69 (63.3%)		1.00
21–25	111	45 (40.5%)		0.40 [0.23,0.68]
26+	86	25 (29.1%)		0.24 [0.13,0.44]
Marital status*			0.051	
Single	216	103 (47.7%)		1.00
Married	75	26 (34.7%)		0.58 [0.34,1.00]
Nationality*			0.620	
Qatari	126	56 (44.4%)		1.00
Other GCC	22	12 (54.5%)		1.50 [0.60,3.73]
Other Arab	128	59 (46.1%)		1.07 [0.65,1.75]
Non-Arab	10	3 (30.0%)		0.54 [0.13,2.17]
Highest level of education			<0.001	
Secondary	157	88 (56.1%)		1.00
University+	149	51 (34.2%)		0.41 [0.26,0.65]
Employment*			0.140	
Yes	84	33 (39.3%)		1.00
No	217	106 (48.8%)		1.48 [0.88,2.46]
Municipality*			0.026	
Doha	155	58 (37.4%)		1.00
Al-Rayan	75	41 (54.7%)		2.02 [1.15,3.53]
Elsewhere	67	34 (50.7%)		1.72 [0.97,3.07]
Household income*			0.430	
1	42	17 (40%)		1.00
2	88	45 (51%)		1.54 [0.73,3.24]
3	63	32 (51%)		1.52 [0.69,3.34]
4	96	40 (42%)		1.05 [0.50,2.20]

^^^Data are presented as n (row %),

^^^^Chi-square test,

^**^^^**^Crude logistic regression,

*1.0–5.9% missing

Regarding the nonprescription supply of antibiotics from pharmacies, 23% of our study population declared that they obtained antibiotics without a prescription from a pharmacy, 12% of whom got them from pharmacy in Qatar (Fig 1). There were no significant associations between demographic factors and nonprescription access to antibiotics from pharmacy (Table 5).

**Table 5:** Association between respondents’ characteristics and obtaining antibiotics from pharmacies without prescription.

	Total N = 581	Obtained antibiotic from pharmacy without prescription^^^ N = 133	p-value^^^^	OR [95% CI] ^^^^^
Participant type*			0.22	1.00
Student	377	80 (21.2%)		1.29 [0.86,1.93]
Family member	198	51 (25.8%)		
Sex*			0.67	1.00
Male	67	17 (25.4%)		0.88 [0.49,1.58]
Female	504	116 (23.0%)		
Age in years			0.28	1.00
<21	224	55 (24.6%)		0.74 [0.47,1.16]
21–25	226	44 (19.5%)		1.08 [0.66,1.77]
26+	131	34 (26.0%)		
Marital status*			0.51	1.00
Single	430	104 (24.2%)		0.85 [0.52,1.38]
Married	122	26 (21.3%)		
Nationality**			0.84	1.00
Qatari	236	52 (22.0%)		1.18 [0.56,2.49]
Other GCC	44	11 (25.0%)		1.04 [0.68,1.61]
Other Arab	237	54 (22.8%)		0.62 [0.18,2.21]
Non-Arab	20	3 (15.0%)		
Highest level of education*			0.28	1.00
Secondary	308	76 (24.7%)		0.81 [0.55,1.19]
University+	273	57 (20.9%)		
Employment*			0.52	1.00
Yes	139	34 (24.5%)		0.86 [0.55,1.35]
No	426	93 (21.8%)		
Municipality*			0.36	1.00
Doha	277	70 (25.3%)		0.71 [0.44,1.14]
Al-Rayan	160	31 (19.4%)		0.95 [0.57,1.58]
Elsewhere	115	28 (24.3%)		
Household income**			0.34	1.00
1	88	18 (20.5%)		1.06 [0.56,2.01]
2	163	35 (21.5%)		1.65 [0.85,3.22]
3	104	31 (29.8%)		1.10 [0.59,2.05]
4	182	40 (22.0%)		1.00

^^^Data are presented as n (row %),

^^^^Chi-square test,

^**^^^**^Crude logistic regression,

*1.0–5.3% missing,

**8.2% missing

### Knowledge on antibiotic use among respondents

**Fig 2:**
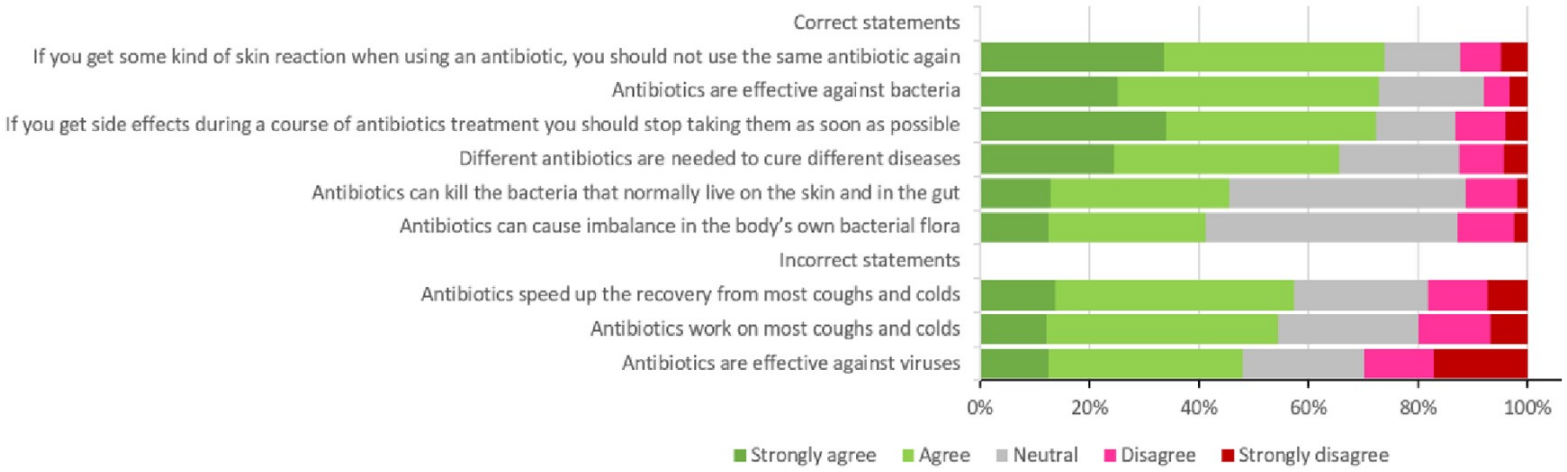
Respondents’ knowledge of antibiotic use.

The questionnaire revealed that respondents had inadequate knowledge about antibiotic use, since the percentage of full agreement (“strongly agree” and “agree”) on the unacceptable knowledge statements was almost 56%. As demonstrated in Fig 2, although the majority of the respondents (73%) believed that antibiotics are effective against bacteria, almost half claimed that antibiotics are effective against viral infections, and approximately 60% of the respondents reported that antibiotics speed up the recovery from most coughs and colds. On the matter of knowledge about antibiotic side effects, only 42% of the respondents reported that antibiotics can cause imbalance in the body’s own bacterial flora, and 46% declared that they can kill the beneficial bacteria that normally live on the skin and in the gut.

The chi-square test results showed that nationality (p = 0.010) and municipality (p = 0.048) were the independent factors associated with having appropriate knowledge of antibiotic use. Approximately 64% of Non-Arab respondents had adequate knowledge about antibiotic use, compared to 41.5% of Qatari respondents. Only 37% of the study population who lived in Al-Rayan municipality had acceptable knowledge of antibiotic use, compared to 45% of those living in Doha. The univariate logistic regression revealed that there were no significant predictors among the demographic factors regarding knowledge of antibiotic use.

### Attitude towards antibiotic use among respondents

**Fig 3:**
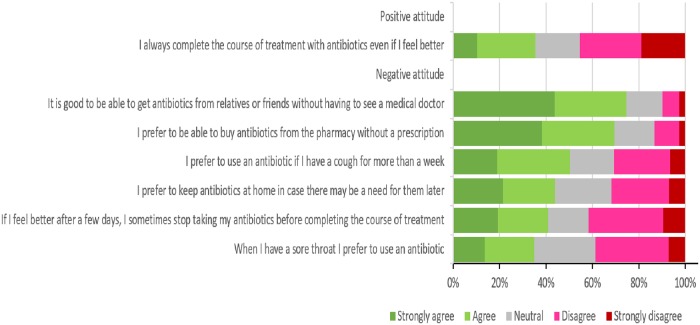
Respondents’ attitude towards antibiotic use.

Regarding the respondents’ attitude towards antibiotic use, the questionnaire also showed that a significant proportion of respondents had a negative attitude towards antibiotic use (59.73%). Interestingly, only 14% of the respondents agreed with the need for a prescription to buy antibiotics from a pharmacy, and 35% declared that they would complete the antibiotics course even if they started to feel better. Three quarters of them claimed that it is good to have the ability to obtain antibiotics from friends or relatives without seeking proper medical advice. Half of the respondents believed that using antibiotics is an effective treatment for coughs, while 35% also believed that it is an appropriate treatment for sore throat (Fig 3).

Chi-square test results revealed that respondents’ attitude towards antibiotic use was significantly associated with age (p = 0.003), marital status (p = 0.010), nationality (p = 0.001) and level of education (p = 0.003). Multivariate analyses demonstrated that older (age>26), married and university-graduated participants had higher odds of having a positive attitude towards antibiotic use than others (OR = 2.06, 95% CI: 1.34–3.17; OR = 1.68, 95% CI: 1.13, 2.51; and OR = 1.66, 95% CI: 1.19–2.31, respectively). Nationality was also significantly associated with respondents’ attitude towards antibiotic use. Compared with Qataris, other GCC respondents had lower odds of having a positive attitude towards antibiotic use (OR = 0.21, 95% CI: 0.09–0.50).

### Respondents’ opinion on healthcare provider antibiotic prescription practices

**Fig 4:**
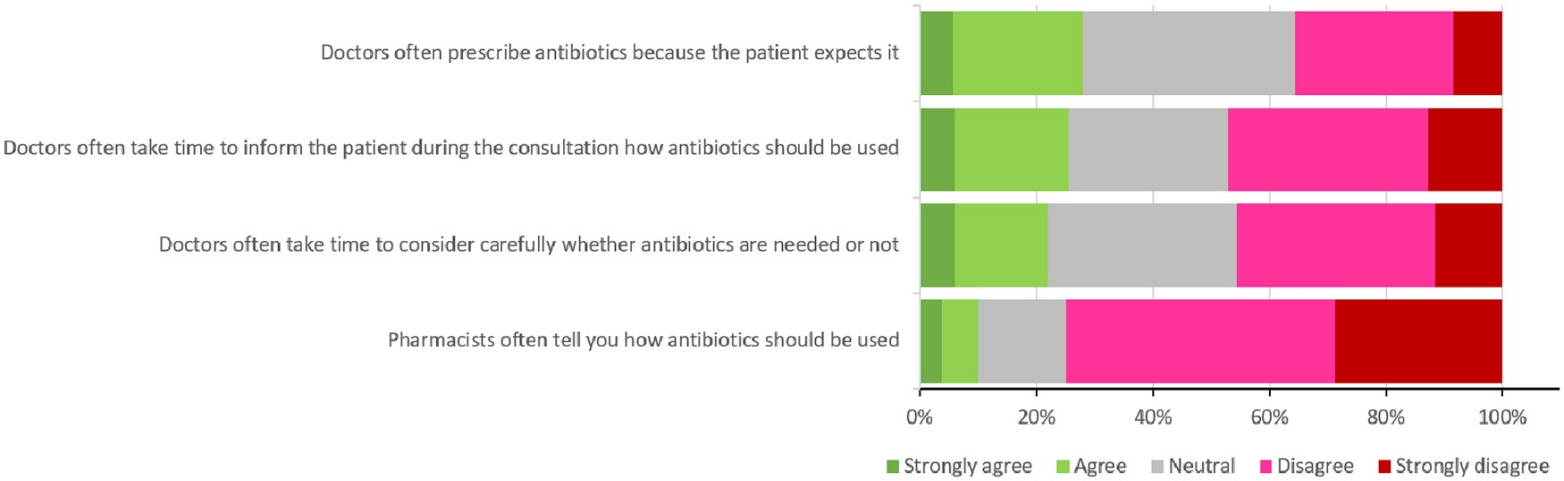
Respondents’ opinions on healthcare providers’ antibiotic prescription practices.

Respondents’ reported that neither doctors nor pharmacists were providing adequate patient education regarding appropriate antibiotic use. Approximately half of the respondents claimed that doctors do not allocate enough time to explain appropriate antibiotic use, and 75% reported the same issue with pharmacists. In addition, 46% of the respondents mentioned that doctors are not cautious when considering whether antibiotics are needed, and 28% reported that doctors often prescribe antibiotics because the patients expect it (Fig 4).

## Discussion

This study aimed to assess the prevalence of inappropriate antibiotic use among Qatar University students and their family members and the different factors associated with this behavior. Our results showed that more than 60% of the respondents had received at least one prescription for antibiotics within the last year prior to our study, which is less than what was reported by a previous study among the public in Qatar (96%) [29], yet still a high percentage. This high rate of prescribing could be explained by patient pressure exerted on physicians to prescribe antibiotics even when they are not needed [35, 36]. Physicians might prescribe antibiotics to mollify patients and maintain a good relationship with them [35, 36]. Previous research conducted in the UK [37] and US [38] pointed to the fact that the patient could influence physicians in antibiotic prescribing, which in turn can cause overprescription. Previous research pointed to the fact that physicians may overestimate patients’ expectations for getting antibiotics and undervalue the necessity of conducting diagnostic tests necessary to confirm the need for antibiotics [39, 40]. A qualitative research study with pharmacists in Qatar revealed that patient pressure to prescribe antibiotic is a major barrier for appropriate antibiotic use [30].

The major inappropriate antibiotic use practices followed by the respondents were using antibiotics without prescription, not completing the antibiotic course, and obtaining antibiotics from a pharmacy without prescription. Our study revealed a high prevalence of inappropriate antibiotic use among the respondents, with 82% reported using antibiotics without prescription, 37% of whom used antibiotics that were prescribed for another family member or someone else and 27% of whom used the same antibiotics that were prescribed for them for a previous illness. A study conducted in Peru in 1000 college students showed that approximately 70% of the participants used antibiotics without medical consultation [41]. A high prevalence of antibiotic self-medication was also reported in other GCC countries [42]. In a very recent systematic review about public knowledge and behaviors relating to antibiotic use in the GCC countries [42], the authors claimed that the overall prevalence of antibiotic self-medication reached 73%, with Saudi Arabia having the highest prevalence [25]. These findings, along with other studies in Qatar [27, 29], indicate the need for public education about the appropriate use of antibiotics and the risks associated with their misuse.

Another finding was about the nonprescription supply of antibiotics from pharmacies: 23% of our study population declared that they obtained antibiotics without a prescription from a pharmacy, 12% of whom got them from a pharmacy in Qatar. Given that people cannot obtain an antibiotic without a medical prescription from pharmacies in Qatar [27], it was surprising to find that, though a small percentage, pharmacies in Qatar are dispensing antibiotics without prescription, as this is against the laws and regulations of the country. This finding is in agreement with another study, which found that 14.2% of the respondents obtained antibiotics without prescription from pharmacies in Qatar despite the current laws [29]. The finding also aligns with other results across the GCC and other Middle Eastern countries. Pharmacies are the main source for antibiotics used in self-medication in the Middle East and a crucial source for antibiotic information. Thus, they are responsible for the misuse, and they need to take a major role in educating patients regarding appropriate antibiotic use [21, 43].

On adherence to the recommended antibiotic regimen, 45% of the population in our study reported that they did not complete their last course of antibiotics. The range of percentages of not completing the course of antibiotics across studies in GCC countries is between 30 and 72, with a 40% median, which may expose the patients to ABR [42]. The meta-analysis and systematic review that included 26 studies from different countries conducted by Gualano et al. showed that 47.1% of the participants stopped taking antibiotic pills once they started feeling better (number) [44]. This practice is attributed to the lack of knowledge about the risks associated with not completing the antibiotic course. The major reasons for not completing the antibiotic course are feeling better and thinking that the antibiotic is not effective in treating the disease [29, 45].

### Sociodemographic variables associated with inappropriate antibiotic use

In our study, nationality and age were associated with using antibiotics without prescription. Qataris and respondents aged less than 21 years reported the highest percentage of inappropriate antibiotic use. The age factor, which was diluted when performing a logistic regression, could be explained by the fact that the young generation does not have appropriate knowledge about antibiotic use. On the contrary, a study reported that older age is a factor negatively affecting the perceived need for antibiotics [46]. As for nationality, Qataris usually travel a lot throughout the year and can easily obtain antibiotics without prescription abroad from countries that have no restrictions on antibiotic purchases from pharmacies. Our findings also revealed that age, level of education and municipality were significant predictors of adherence to the antibiotic course. Respondents less than 21 years of age were more likely to stop taking the antibiotic before completing the course. Moreover, respondents who had secondary education were less likely to complete their antibiotic courses compared to the higher-education group. Respondents who lived in Al-Rayan municipality were 2.4 times more likely to stop their antibiotic course early compared to those who lived in Doha. This indicates that in those areas in Qatar, people have inadequate education about antibiotic use. An explanation for this finding could be that primary health care centers in these areas are overwhelmed with the number of patients they receive, which may hinder health care providers in providing adequate patient education and lead patients to follow inappropriate antibiotic practices.

### Individual factors: Knowledge and attitude towards antibiotic use

Our study revealed that the respondents had inadequate knowledge about antibiotic use, since the percentage of their full agreement (“strongly agree” and “agree”) on the unacceptable knowledge statements was almost 56%. The majority of the respondents (73%) believed that antibiotics are effective against bacteria; almost half claimed that antibiotics are effective against viral infections, and approximately 60% of the respondents reported that antibiotics speed up the recovery from most coughs and colds. A study reported that although approximately 70% of the participants acknowledged the value of antibiotics in treating bacterial infections, half of the participants thought that they could also use antibiotics for non-bacterial infections [42]. Moienzadeh and his colleagues also showed that there are gaps in the knowledge about antibiotic use in Qatar, as 47% believed that antibiotics are effective against viral infections and 40% believed that not taking an antibiotic would delay the recovery from coughs and colds [29]. The meta-analysis and systematic review conducted by Gualano et al. on the population’s knowledge and attitude about antibiotics showed a lack of knowledge. Specifically, 33.7% of the subjects did not know that antibiotics are useful to treat bacterial infections, and 53.9% of them did not know that antibiotics cannot treat viral infections [44]. Concerning knowledge about antibiotics’ side effects, only 42% of the respondents in our study reported that antibiotics can cause imbalance in the body’s own bacterial flora, and 46% declared that antibiotics can kill the beneficial bacteria that normally live on the skin and in the gut. Slight higher results regarding public knowledge about the side effects of antibiotics were reported by a Kuwaiti study [34]. Gualano and his colleagues showed that 59.4% of the subjects in the systematic review were aware of antibiotic resistance. However, 26.9% of them did not know that misuse of antibiotics can lead to antibiotic resistance [44].

The chi-square test results in our study showed that nationality and municipality were the independent factors associated with having appropriate knowledge of antibiotic use. This reinforces our previous findings that Qatari respondents living in Doha have inadequate knowledge about antibiotic use. Results from antibiotic awareness surveys have shown that although individuals would have good knowledge of when antibiotics should be used, they would still obtain antibiotics without prescription [47–49]. Thus, it is not only the level of knowledge regarding antibiotic use that may influence people’s practices, but their attitude regarding antibiotic use also has a large role in shaping these practices [34]. A significant proportion of the respondents had a negative attitude towards antibiotic use (59.73%) in this study. Only 35% declared that they would complete the antibiotic course even if they started to feel better. Previous research in Qatar showed that one-third of participants stop taking antibiotic pills once they feel better [29]. Univariate logistic regression analyses in our study demonstrated that older (age>26), married and university-graduated participants were more likely to have a positive attitude towards antibiotic use than others, which also stresses the importance of the age factor regarding antibiotic use. Married people usually have more experience with infections, as they usually deal with children. Education also seems to be an important factor in regard to antibiotic use.

### Social network: Family and friends

Previous research points to the crucial role that family members play as sources of antibiotic information, recommendations and supply [34]. Qatar has a family-centered culture. In our study, 37% of the respondents who reported using antibiotics without prescription admitted using antibiotics that were prescribed for another family member or someone else. Moienzadeh and his colleagues reported the same percentage when they conducted their study at a community pharmacy in Qatar, where patients admitted giving their antibiotics to a family member if they developed an infection [29]. In addition, three-quarters of them reported a negative attitude, showing that it is fine to obtain antibiotics from relatives and friends without seeking proper medical advice.

### Organizational factors: Role of healthcare providers

The last finding is about the respondents’ opinion regarding the healthcare providers’ antibiotic prescription practices. Respondents reported that neither doctors nor pharmacists provide adequate patient education about appropriate antibiotic use. Approximately half of the respondents claimed that physicians do not allocate enough time to explain appropriate antibiotic use, and 75% reported the same issue with pharmacists. A recent Australian study reported that detailed information about appropriate antibiotic use was not voluntarily provided by either the GP or community pharmacist if not asked for by the patients [50], and sometimes patients sought other sources of information. Hawke and his colleagues, in their retrospective analysis of an Australian medicine helpline, reported that 40% of antibiotic-related calls were due to inadequate information provision [51]. Shaikhan et al reported, in their systematic review, that 97.3% of participants across the studies in Qatar stated that physicians’ explanations are helpful, and 88.2% feel comfortable if treatment was discussed [27]. These numbers reinforce the need to enhance the quality of patient-provider communication to explain appropriate antibiotic use. In addition, 46% of the respondents in our study mentioned that doctors are not cautious when considering whether antibiotics are needed, and 28% reported that doctors often prescribe antibiotics because the patients expect it. Previous studies reported that physicians prescribe antibiotics just to satisfy patients even when they are not needed, and almost half of the patients in the studies reviewed by Shaikhan and his colleagues would feel dissatisfied when not prescribed an antibiotic [21].

Any effort to tackle antibiotic resistance must include patient education and counseling, as patient demands are a major driver of overuse and misuse of antibiotics. Health organizations must consider social media within their communication strategy to promote appropriate antibiotic-related information seeking in the general population. A recent study demonstrated that the Internet is a resource for disseminating quality health information that has the potential to improve the stewardship of antibiotics in the community [52]. Anderson reported that the Internet as a source of information about antibiotics was independently and positively associated with knowledge, attitude, and behavior regarding antibiotics [23].

Our study has some strengths. To the best of our knowledge, this study is the first to assess inappropriate antibiotic use practices among university students and their family members and the factors influencing this behavior in the State of Qatar. Understanding how some factors shaping public practices of antibiotic use are embedded in the different layers of the ecological model would help address the behavior comprehensively and tailor the findings towards evidence-based, multifactorial, effective interventions.

Study limitations: The limitations of this study include the cross-sectional design, which limits our ability to infer causation between the various factors and antibiotic use practices. The small sample size and the use of convenience sampling, which may not truly represent the overall population being studied (e.g., the adult population older than 26 years is underrepresented), limits the generalization of the findings to the study population level. Other potential limitations include the social desirability bias of respondents, which may have led them to offer favorable answers, and the use of a self-administered survey, which may have led to recall bias.

An Action Plan was developed in Qatar to reduce the inappropriate antibiotic use that causes the spread of ABR. The Action Plan is supposed to implement antimicrobial stewardship in all healthcare settings, aiming to improve public antibiotic use through promotion programs and interventions [27]. A few studies on antibiotic use have been conducted to explore people’s knowledge, attitude and practice regarding antibiotics in Qatar. The current study addresses a gap in the literature regarding sociodemographic, individual, and organizational factors influencing public practices of antibiotic use in Qatar. Understanding these factors will provide evidence for health educators and policy makers to plan for effective programs that promote public practices of antibiotic use, which in turn will decrease the prevalence of ABR. Future research should focus on the determinants associated with inappropriate antibiotic use with a representative sample of the public in the country. More qualitative research is also needed to study in depth the antibiotic prescription process followed by physicians and pharmacists in primary health care and the factors shaping the prescription process.

## Conclusion

This study showed that antibiotic use is a multifactorial behavior shaped by demographic, individual (knowledge and attitude), and organizational factors. Young Qatari individuals with a secondary educational who live in large and crowded cities in Qatar (Doha and Al Rayan) are at high risk of following inappropriate antibiotic use practices, such as using antibiotics without prescription, not completing the antibiotic course, and obtaining antibiotics from a pharmacy without prescription. Health educators should design awareness campaigns to educate the public, especially young adults, about the appropriate antibiotic use practices and the consequences of their misuse. Educational sessions can focus on school children and adolescents. Sharing educational messages through social media about appropriate antibiotic use, the difference between bacterial and viral infections and how antibiotics should not be used for viral infections, antibiotics’ side effects, and ABR could be an effective health promotion method for the young generation. Awareness campaigns should involve different family members since family is considered an important source of both antibiotics and health information regarding antibiotic use. Health educators should work not only on providing knowledge but also on changing the attitudes and behaviors regarding antibiotic use through different behavioral interventions.

At the organizational level, the healthcare system in the public and private sectors should offer training for professionals who have the authority to prescribe antibiotics, such as physicians and pharmacists, to enhance awareness about antibiotic prescribing guidelines and the patient- health care provider communication process regarding appropriate antibiotic use. There should be an emphasis on applying the guidelines and laws when prescribing and dispensing antibiotics.

## Supporting information

S1 Questionnaire English questionnaire. (DOCX)

S2 Questionnaire Arabic questionnaire. (DOCX)

## References

[1] World Health Organization. Antibiotic resistance 2018 https://www.who.int/news-room/fact-sheets/detail/antibiotic-resistance.

[2] Davies DS VE. Antimicrobial Resistance: In Search of a Collaborative Solution.Report of the Antimicrobial Resistance Working Group 2013.; 2013.

[3] Centers for Disease Control and Prevention. About antimicrobial resistance 2018 https://www.cdc.gov/drugresistance/about.html?fbclid=IwAR1MiptNr4RaP1nsekpUJWiFQbtHUMCsl8KXZsuxEVcgT5-hftjbDXF4rsI.

[4] Mackenbach JP, Looman CW. Secular trends of infectious disease mortality in The Netherlands, 1911–1978: quantitative estimates of changes coinciding with the introduction of antibiotics. Int J Epidemiol. 1988;17(3):618–24. 10.1093/ije/17.3.618 3209342

[5] Centers for Disease Control and Prevention. Antibiotic resistance threat in the United States. Atlanta, GA: US Department of Health and Human Services 2013.

[6] Cassini A, Hogberg LD, Plachouras D, Quattrocchi A, Hoxha A, Simonsen GS, et al Attributable deaths and disability-adjusted life-years caused by infections with antibiotic-resistant bacteria in the EU and the European Economic Area in 2015: a population-level modelling analysis. Lancet Infect Dis. 2019;19(1):56–66. 10.1016/S1473-3099(18)30605-4 30409683 PMC6300481

[7] Memish ZA, Ahmed QA, Arabi YM, Shibl AM, Niederman MS. Microbiology of community-acquired pneumonia in the Gulf Corporation Council states. J Chemother. 2007;19 Suppl 1:17–23. 10.1080/1120009x.2007.11782430 18073166

[8] Aly M, Balkhy HH. The prevalence of antimicrobial resistance in clinical isolates from Gulf Corporation Council countries. Antimicrob Resist Infect Control. 2012;1(1):26 10.1186/2047-2994-1-26 22958584 PMC3436690

[9] Zhang R, Eggleston K, Rotimi V, Zeckhauser RJ. Antibiotic resistance as a global threat: evidence from China, Kuwait and the United States. Global Health. 2006;2:6 10.1186/1744-8603-2-6 16603071 PMC1502134

[10] Alhomoud F, Aljamea Z, Basalelah L. "Antibiotics kill things very quickly"—consumers’ perspectives on non-prescribed antibiotic use in Saudi Arabia. BMC public health. 2018;18(1):1177 10.1186/s12889-018-6088-z 30326870 PMC6192199

[11] Costelloe C, Metcalfe C, Lovering A, Mant D, Hay AD. Effect of antibiotic prescribing in primary care on antimicrobial resistance in individual patients: systematic review and meta-analysis. Bmj. 2010;340:c2096 10.1136/bmj.c2096 20483949

[12] Bell BG, Schellevis F, Stobberingh E, Goossens H, Pringle M. A systematic review and meta-analysis of the effects of antibiotic consumption on antibiotic resistance. BMC Infect Dis. 2014;14:13 10.1186/1471-2334-14-13 24405683 PMC3897982

[13] CDC. Improve Antibiotic Prescribing 2019 https://www.cdc.gov/drugresistance/protecting_patients.html.

[14] Shapiro DJ, Hicks LA, Pavia AT, Hersh AL. Antibiotic prescribing for adults in ambulatory care in the USA, 2007–09. J Antimicrob Chemother. 2014;69(1):234–40. 10.1093/jac/dkt301 23887867

[15] Bianco A, Papadopoli R, Mascaro V, Pileggi C, Pavia M. Antibiotic prescriptions to adults with acute respiratory tract infections by Italian general practitioners. Infect Drug Resist. 2018;11:2199–205. 10.2147/IDR.S170349 30519057 PMC6233949

[16] Lindberg BH, Gjelstad S, Foshaug M, Hoye S. Antibiotic prescribing for acute respiratory tract infections in Norwegian primary care out-of-hours service. Scand J Prim Health Care. 2017;35(2):178–85. 10.1080/02813432.2017.1333301 28569649 PMC5499318

[17] Goggin K, Bradley-Ewing A, Myers AL, Lee BR, Hurley EA, Delay KB, et al Protocol for a randomised trial of higher versus lower intensity patient-provider communication interventions to reduce antibiotic misuse in two paediatric ambulatory clinics in the USA. BMJ Open. 2018;8(5):e020981 29743330 10.1136/bmjopen-2017-020981 PMC5942422

[18] Currie J, Lin W, Zhang W. Patient knowledge and antibiotic abuse: Evidence from an audit study in China. J Health Econ. 2011;30(5):933–49. 10.1016/j.jhealeco.2011.05.009 21733587

[19] Zwisler G, Simpson E, Moodley M. Treatment of diarrhea in young children: results from surveys on the perception and use of oral rehydration solutions, antibiotics, and other therapies in India and Kenya. J Glob Health. 2013;3(1):010403-. 10.7189/jogh.03.010403 23826507 PMC3700033

[20] Al-Niemat SI, Bloukh DT, Al-Harasis MD, Al-Fanek AF, Salah RK. Drug use evaluation of antibiotics prescribed in a Jordanian hospital outpatient and emergency clinics using WHO prescribing indicators. Saudi Med J. 2008;29(5):743–8. 18454225

[21] Alhomoud F, Aljamea Z, Almahasnah R, Alkhalifah K, Basalelah L, Alhomoud FK. Self-medication and self-prescription with antibiotics in the Middle East-do they really happen? A systematic review of the prevalence, possible reasons, and outcomes. International journal of infectious diseases: IJID: official publication of the International Society for Infectious Diseases. 2017;57:3–12. 28111172 10.1016/j.ijid.2017.01.014

[22] Napolitano F, Izzo MT, Di Giuseppe G, Angelillo IF. Public knowledge, attitudes, and experience regarding the use of antibiotics in Italy. PLoS One. 2013;8(12):e84177 10.1371/journal.pone.0084177 24376793 PMC3871686

[23] Anderson A. Online health information and public knowledge, attitudes, and behaviours regarding antibiotics in the UK: Multiple regression analysis of Wellcome Monitor and Eurobarometer Data. PLoS One. 2018;13(10):e0204878 10.1371/journal.pone.0204878 30356302 PMC6200201

[24] Bert F, Gualano MR, Gili R, Scaioli G, Lovato E, Angelillo IF, et al Knowledge and attitudes towards the use of antibiotics in the paediatric age group: a multicenter survey in Italy. Eur J Public Health. 2017;27(3):506–12. 10.1093/eurpub/ckw209 27836969

[25] Hawkings NJ, Butler CC, Wood F. Antibiotics in the community: a typology of user behaviours. Patient Educ Couns. 2008;73(1):146–52. 10.1016/j.pec.2008.05.025 18640805

[26] Morgan DJ, Okeke IN, Laxminarayan R, Perencevich EN, Weisenberg S. Non-prescription antimicrobial use worldwide: a systematic review. Lancet Infect Dis. 2011;11(9):692–701. 10.1016/S1473-3099(11)70054-8 21659004 PMC3543997

[27] Shaikhan F, Rawaf S, Majeed A, Hassounah S. Knowledge, attitude, perception and practice regarding antimicrobial use in upper respiratory tract infections in Qatar: a systematic review. JRSM open. 2018;9(9):2054270418774971. 10.1177/2054270418774971 PMC6122249 30202533

[28] Alkhuzaei AMJB, Salama RE, Eljak IEI, Chehab MA, Selim NA. Perceptions and practice of physicians and pharmacists regarding antibiotic misuse at primary health centres in Qatar: A cross-sectional study. Journal of Taibah University Medical Sciences. 2018;13(1):77–82. 10.1016/j.jtumed.2017.09.001 31435306 PMC6694943

[29] Moienzadeh A, Massoud T, Black E. Evaluation of the general public’s knowledge, views and practices relating to appropriate antibiotic use in Qatar. Int J Pharm Pract. 2017;25(2):133–9. 10.1111/ijpp.12233 26671211

[30] Black E, Cartwright A, Bakharaiba S, Al-Mekaty E, Alsahan D. A qualitative study of pharmacists’ perceptions of, and recommendations for improvement of antibiotic use in Qatar. International journal of clinical pharmacy. 2014;36(4):787–94. 10.1007/s11096-014-9960-7 24899213

[31] Emeka PM, Al-Omar M, Khan TM. Public attitude and justification to purchase antibiotics in the Eastern region Al Ahsa of Saudi Arabia. Saudi Pharm J. 2014;22(6):550–4. 10.1016/j.jsps.2014.02.014 25561868 PMC4281610

[32] Epi info software version 7. 2018 https://www.cdc.gov/epiinfo/pc.html.

[33] World Health Organization. ANTIBIOTIC RESISTANCE: MULTI-COUNTRY PUBLIC AWARENESS SURVEY. 2015.

[34] Awad AI, Aboud EA. Knowledge, attitude and practice towards antibiotic use among the public in Kuwait. PLoS One. 2015;10(2):e0117910–e. 10.1371/journal.pone.0117910 25675405 PMC4326422

[35] Steinman MA, Landefeld CS, Gonzales R. Predictors of broad-spectrum antibiotic prescribing for acute respiratory tract infections in adult primary care. Jama. 2003;289(6):719–25. 10.1001/jama.289.6.719 12585950

[36] Little P, Dorward M, Warner G, Stephens K, Senior J, Moore M. Importance of patient pressure and perceived pressure and perceived medical need for investigations, referral, and prescribing in primary care: nested observational study. Bmj. 2004;328(7437):444 10.1136/bmj.38013.644086.7C 14966079 PMC344266

[37] Butler CC, Rollnick S, Pill R, Maggs-Rapport F, Stott N. Understanding the culture of prescribing: qualitative study of general practitioners' and patients' perceptions of antibiotics for sore throats. BMJ. 1998;317(7159):637 10.1136/bmj.317.7159.637 9727992 PMC28658

[38] Ong S, Nakase J, Moran GJ, Karras DJ, Kuehnert MJ, Talan DA. Antibiotic use for emergency department patients with upper respiratory infections: prescribing practices, patient expectations, and patient satisfaction. Ann Emerg Med. 2007;50(3):213–20. 10.1016/j.annemergmed.2007.03.026 17467120

[39] Institute of Medicine Forum on Emerging I. The National Academies Collection: Reports funded by National Institutes of Health In: Knobler SL, Lemon SM, Najafi M, Burroughs T, editors. The Resistance Phenomenon in Microbes and Infectious Disease Vectors: Implications for Human Health and Strategies for Containment: Workshop Summary. Washington (DC): National Academies Press (US) National Academy of Sciences; 2003. 22649806

[40] World health organization. WHO global strategy for containment of antimicrobial resistance. 2001.

[41] Núñez M, Tresierra-Ayala M, Gil-Olivares F. Antibiotic self-medication in university students from Trujillo, Peru. Medicina Universitaria. 2016;18(73):205–9.

[42] Almohammed RA, Bird EL. Public knowledge and behaviours relating to antibiotic use in Gulf Cooperation Council countries: A systematic review. J Infect Public Health. 2019;12(2):159–66. 10.1016/j.jiph.2018.09.002 30245158

[43] Scicluna EA, Borg MA, Gur D, Rasslan O, Taher I, Redjeb SB, et al Self-medication with antibiotics in the ambulatory care setting within the Euro-Mediterranean region; results from the ARMed project. J Infect Public Health. 2009;2(4):189–97. 10.1016/j.jiph.2009.09.004 20701882

[44] Gualano MR, Gili R, Scaioli G, Bert F, Siliquini R. General population’s knowledge and attitudes about antibiotics: a systematic review and meta-analysis. Pharmacoepidemiology and drug safety. 2015;24(1):2–10. 10.1002/pds.3716 25251203

[45] Bin Nafisah S, Bin Nafesa S, Alamery AH, Alhumaid MA, AlMuhaidib HM, Al-Eidan FA. Over-the-counter antibiotics in Saudi Arabia, an urgent call for policy makers. J Infect Public Health. 2017;10(5):522–6. 10.1016/j.jiph.2016.09.016 28162966

[46] Bashwar Z, Malik MA, Fawaz M, Said H. Knowledge, Attitudes and Practices of Patients Attending West Bay Health Center Towards Upper Respiratory Tract Infections. Qatar Medical Journal. 2006;2006(1).

[47] Huttner B, Goossens H, Verheij T, Harbarth S. Characteristics and outcomes of public campaigns aimed at improving the use of antibiotics in outpatients in high-income countries. Lancet Infect Dis. 2010;10(1):17–31. 10.1016/S1473-3099(09)70305-6 20129146

[48] Saam M, H B, Harbarth S. Evaluation of Antibiotic Awareness Campaigns. Geneva, Switzerland: WHO Collaborating Centre on Patient Safety, 2017.

[49] McNulty CA, Boyle P, Nichols T, Clappison P, Davey P. Don’t wear me out—the public’s knowledge of and attitudes to antibiotic use. J Antimicrob Chemother. 2007;59(4):727–38. 10.1093/jac/dkl558 17307770

[50] Lum EPM, Page K, Nissen L, Doust J, Graves N. Australian consumer perspectives, attitudes and behaviours on antibiotic use and antibiotic resistance: a qualitative study with implications for public health policy and practice. BMC public health. 2017;17(1):799 10.1186/s12889-017-4813-7 29017473 PMC5635573

[51] Hawke KL, McGuire TM, Ranmuthugala G, van Driel ML. What do consumers want to know about antibiotics? Analysis of a medicines call centre database. Fam Pract. 2016;33(1):75–81. 10.1093/fampra/cmv083 26502809

[52] Zucco R, Lavano F, Anfosso R, Bianco A, Pileggi C, Pavia M. Internet and social media use for antibiotic-related information seeking: Findings from a survey among adult population in Italy. Int J Med Inform. 2018;111:131–9. 10.1016/j.ijmedinf.2017.12.005 29425624

