# Supplementary material for: Public practices on antibiotic use: A cross-sectional study among Qatar University students and their family members
Source: PLoS One. 2019 Nov 26;14(11):e0225499. doi: 10.1371/journal.pone.0225499 (PMC6879134; doi:10.1371/journal.pone.0225499)
Supplement: S1 Questionnaire — (DOCX) [file pone.0225499.s001.docx]

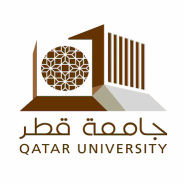

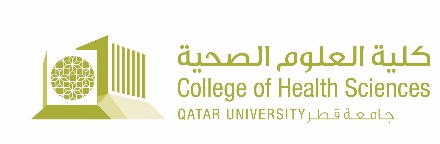
**Questionnaire**

The purpose of this study is to explore the current public practices on antibiotic usage among Qatar University students and their family members. After you agree to participate in our study and sign a consent form, please fill this questionnaire and return back to the collector. The collector will handle another questionnaire for you to be filled by a family member.

| **Q1. ID ___ ___ ___** | **Q2. Date: (DD/MM/YYYY):___ ___ / ___ ___ / ___ ___ ___ ___** | | |
| --- | --- | --- | --- |
| Socio-demographic characteristics DM | | | |
| Q3. Are you QU student? | | Yes 1  No 2 | 1 ⇨ Q5 |
| Q4. What is your relationship to QU student? | | Mother 1  Father 2  Sister 3  Brother 4  Others (Specify) 8 |  |
| Q5. What is your gender? | | Male 1  Female 2 |  |
| Q6.What is your age in years? | | Age in years ____ ____ |  |
| Q7. What is your marital status? | | Single 1  Married 2  Others (Specify) 8 |  |
| Q8. What is your Nationality? | | Qatari 1  Other GCC 2  Other Arab 3  Subcontinent Indian 4  South East Asian 5  Non Arab African 6 |  |
| Q9. What is the highest educational level you completed? | | Primary 1  Secondary 2  University or higher 3  Not educated 4 |  |
| Q10. Are you employed? | | Yes 1  No 2 | 2 ⇨ Q12 |
| Q11. Where are you employed? | | Health-related organization 1  Non health–related organization 2  Others (Specify) 8 |  |
| Q12. What is your area of residence? | | Doha 1  Al Rayyan 2  Alkhour 3  Alwakra…………………………………………………………. 4  Alshamal 5  Umm Salal 6  Adaghaein 7 |  |
| Q13. Which of following best describes your total household income? | | Less than 10,000 QR 1  10,000-20,000 QR 2  20,000-30,000 QR 3  More than 30,000 QR 4 |  |
| Q14. What is your gender? | | Male 1  Female 2 |  |
| **Antibiotic usAge** | | | |
| AU1. Have you been prescribed an antibiotic within the last year? | | Yes 1  No 2  Do not know 6 | 2 ⇨ AU5 |
| AU2. How many times have you been prescribed an antibiotic during the last year? | | None 1  Once 2  Twice 3  Three times 4  More than three times 5  Do not know 6 |  |
| AU3. Did you complete your last antibiotic course as being prescribed? | | Yes 1  No 2  Do not know 6 | 1 ⇨ AU5 |
| AU4. Why you Did not complete the course? | | Was cured before the end of the course 1  Forgot to take the pills 2  Had side effects from the drug and was advised by a physician to stop taking it 3  Was advised by a relative/friend to stop taking the drug 4  Others (Specify) 8 |  |
| AU5. Have you used an antibiotic **without** being prescribed by a doctor or dentist within the last year (self-medication)? | | Yes 1  No 2  Do not know 6 | 2 ⇨ AU8 |
| AU6. How many times have you used self-medication with an antibiotic during the last year? | | None 1  Once 2  Twice 3  Three times 4  More than three times 5  Do not know 6 |  |
| AU7. What was the condition(s) for which the antibiotic has been used without prescription? | | Headache 1  Body ache 2  Sore throat…………………………………………………….3  Skin or wound infection 4  Fever……………………………………………………………..5  Cold and flu 6  Diarrhea 7  Urinary tract infection 8  Others (Specify) 8 |  |
| AU8. Did you finish your last antibiotic course as being prescribed? | | Yes 1  No 2  Do not know 6 |  |
| AU9. Have you ever used antibiotics that were originally prescribed for an infection which recurred later? | | Yes 1  No 2  Do not know ………………………………………… 6 |  |
| AU10. Have you ever used an originally prescribed antibiotic for another type of infection? | | Yes 1  No 2  Do not know ………………………………………… 6 |  |
| AU11. Have you ever obtained an antibiotic from a pharmacy abroad without a prescription? | | Yes 1  No 2  Do not know ………………………………………… 6 |  |
| AU12. Have you ever obtained an antibiotic from a pharmacy within Qatar without a prescription? | | Yes 1  No 2  Do not know ………………………………………… 6 |  |
| AU13. Have you ever used an antibiotic originally prescribed for another family member? | | Yes 1  No 2  Do not know ………………………………………… 6 |  |
| AU14. Have you ever used an antibiotic originally prescribed for someone else who was not a family member? | | Yes 1  No 2  Do not know ………………………………………… 6 |  |

| **Antibiotic usAge: Do you agree with the following statements or not?** | | |
| --- | --- | --- |
| AG15.Different antibiotics are needed to cure different diseases. | Strongly disagree……………………………………………1  Disagree………………………………………………………….2  Neutral……………………………………………………………3  Agree………………………………………………………………4  Strongly agree…………………………………………………5 |  |
| AG16. ANTIBIOTICS ARE EFFECTIVE AGAINST BACTERIA. | Strongly disagree……………………………………………1  Disagree………………………………………………………….2  Neutral……………………………………………………………3  Agree………………………………………………………………4  Strongly agree…………………………………………………5 |  |
| AG17.ANTIBIOTICS CAN KILL THE BACTERIA THAT NORMALLY LIVE ON THE SKIN AND IN THE GUT. | Strongly disagree……………………………………………1  Disagree………………………………………………………….2  Neutral……………………………………………………………3  Agree………………………………………………………………4  Strongly agree…………………………………………………5 |  |
| AG18.ANTIBIOTICS SPEED UP THE RECOVERY FROM MOST COUGHS AND COLDS. | Strongly disagree……………………………………………1  Disagree………………………………………………………….2  Neutral……………………………………………………………3  Agree………………………………………………………………4  Strongly agree…………………………………………………5 |  |
| AG19.ANTIBIOTICS WORK ON MOST COUGHS AND COLDS. | Strongly disagree……………………………………………1  Disagree………………………………………………………….2  Neutral……………………………………………………………3  Agree………………………………………………………………4  Strongly agree…………………………………………………5 |  |
| AG20. ANTIBIOTICS ARE EFFECTIVE AGAINST VIRUSES. | Strongly disagree……………………………………………1  Disagree………………………………………………………….2  Neutral……………………………………………………………3  Agree………………………………………………………………4  Strongly agree…………………………………………………5 |  |
| AG21. IF YOU GET SIDE EFFECTS DURING A COURSE OF ANTIBIOTICS TREATMENT YOU SHOULD STOP TAKING THEM AS SOON AS POSSIBLE. | Strongly disagree……………………………………………1  Disagree………………………………………………………….2  Neutral……………………………………………………………3  Agree………………………………………………………………4  Strongly agree…………………………………………………5 |  |
| AG22. IF YOU GET SOME KIND OF SKIN REACTION WHEN USING AN ANTIBIOTIC, YOU SHOULD NOT USE THE SAME ANTIBIOTIC AGAIN. | Strongly disagree……………………………………………1  Disagree………………………………………………………….2  Neutral……………………………………………………………3  Agree………………………………………………………………4  Strongly agree…………………………………………………5 |  |
| AG23. ANTIBIOTICS CAN CAUSE IMBALANCE IN THE BODY’S OWN BACTERIAL FLORA. | Strongly disagree……………………………………………1  Disagree………………………………………………………….2  Neutral……………………………………………………………3  Agree………………………………………………………………4  Strongly agree…………………………………………………5 |  |
| AG24. I ALWAYS COMPLETE THE COURSE OF TREATMENT WITH ANTIBIOTICS EVEN IF I FEEL BETTER. | Strongly disagree……………………………………………1  Disagree………………………………………………………….2  Neutral……………………………………………………………3  Agree………………………………………………………………4  Strongly agree…………………………………………………5 |  |
| AG25. IT IS GOOD TO BE ABLE TO GET ANTIBIOTICS FROM RELATIVES OR FRIENDS WITHOUT HAVING TO SEE A MEDICAL DOCTOR. | Strongly disagree……………………………………………1  Disagree………………………………………………………….2  Neutral……………………………………………………………3  Agree………………………………………………………………4  Strongly agree…………………………………………………5 |  |
| AG26. I PREFER TO BE ABLE TO BUY ANTIBIOTICS FROM THE PHARMACY WITHOUT A PRESCRIPTION. | Strongly disagree……………………………………………1  Disagree………………………………………………………….2  Neutral……………………………………………………………3  Agree………………………………………………………………4  Strongly agree…………………………………………………5 |  |
| AG27. I PREFER TO KEEP ANTIBIOTICS AT HOME IN CASE THERE MAY BE A NEED FOR THEM LATER. | Strongly disagree……………………………………………1  Disagree………………………………………………………….2  Neutral……………………………………………………………3  Agree………………………………………………………………4  Strongly agree…………………………………………………5 |  |
| AG28. IF I FEEL BETTER AFTER A FEW DAYS, I SOMETIMES STOP TAKING MY ANTIBIOTICS BEFORE COMPLETING THE COURSE OF TREATMENT. | Strongly disagree……………………………………………1  Disagree………………………………………………………….2  Neutral……………………………………………………………3  Agree………………………………………………………………4  Strongly agree…………………………………………………5 |  |
| AG29. I PREFER TO USE AN ANTIBIOTIC IF I HAVE A COUGH FOR MORE THAN A WEEK. | Strongly disagree……………………………………………1  Disagree………………………………………………………….2  Neutral……………………………………………………………3  Agree………………………………………………………………4  Strongly agree…………………………………………………5 |  |
| AG30. WHEN I HAVE A SORE THROAT I PREFER TO USE AN ANTIBIOTIC. | Strongly disagree……………………………………………1  Disagree………………………………………………………….2  Neutral……………………………………………………………3  Agree………………………………………………………………4  Strongly agree…………………………………………………5 |  |
| **Healthcare providers and antibiotic usage** |  |  |
| HC1. Pharmacists often tell you how antibiotics should be used. | Strongly disagree……………………………………………1  Disagree………………………………………………………….2  Neutral……………………………………………………………3  Agree………………………………………………………………4  Strongly agree…………………………………………………5 |  |
| HC2. Doctors often take time to inform the patient during the consultation how antibiotics should be used. | Strongly disagree……………………………………………1  Disagree………………………………………………………….2  Neutral……………………………………………………………3  Agree………………………………………………………………4  Strongly agree…………………………………………………5 |  |
| HC3. Doctors often prescribe antibiotics because the patient expects it. | Strongly disagree……………………………………………1  Disagree………………………………………………………….2  Neutral……………………………………………………………3  Agree………………………………………………………………4  Strongly agree…………………………………………………5 |  |
| HC4. Doctors often take time to consider carefully whether antibiotics are needed or not. | Strongly disagree……………………………………………1  Disagree………………………………………………………….2  Neutral……………………………………………………………3  Agree………………………………………………………………4  Strongly agree…………………………………………………5 |  |

Thank you!
