# Supplementary material for: Public practices on antibiotic use: A cross-sectional study among Qatar University students and their family members
Source: PLoS One. 2019 Nov 26;14(11):e0225499. doi: 10.1371/journal.pone.0225499 (PMC6879134; doi:10.1371/journal.pone.0225499)
Supplement: S2 Questionnaire — (DOCX) [file pone.0225499.s002.docx]

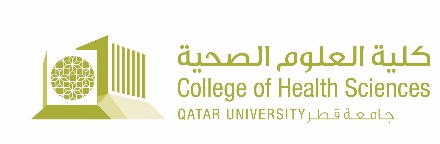

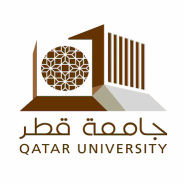


**استبيان**

ان الغرض من هذا البحث هو استكشاف تاثير السلوكيات / الممارسات العامة الحالية على استخدام المضادات الحيوية بين طلاب جامعة قطر وأفراد أسرهم. . سيقوم الطلاب بملء الاستبيان وإعادته إلى جامعي البيانات. كما سيتم اعطاء كل مشارك استمارة موافقة قبل ملء الاستبيان واستمارة آخرى لفرد من الأسرة. كما ستتاح للمشاركين الفرصة لطرح أي سؤال قد يكون لديهم..

| **Q1. معرف:Q2 . تاريخ: (يوم / شهر/ سنة): ___ ___ / ___ ___ / ___ ___ ___ ___** | | | |
| --- | --- | --- | --- |
| الخصائص الاجتماعية والديموغرافية | | | |
| 1 ⇨ Q5 | نعم......................................................1 لا........................................................2 | هل أنت طالب في جامعة قطر؟ | .Q3 |
|  | ام........................................................1 اب.......................................................2 اخت.....................................................3  اخ........................................................4  أخرى (حدد)............................................8 | ما هي علاقتك بطالب الجامعه ؟ | .Q4 |
|  | ذكر......................................................1  انثى......................................................2 | ما هو جنسك؟ | .Q5 |
|  | العمر بالسنين ______ ______ | ما هو عمرك بالسنوات؟ | .Q6 |
|  | عازب...................................................1  متزوج...................................................2  أخرى (حدد)............................................8 | ما هو وضعك العائلي؟ | .Q7 |
|  | قطر......................................................1  دول مجلس التعاون الخليجي الأخرى.................2  عربي آخر..............................................3  شبه القارة الهندية.......................................4  جنوب شرق آسيا.......................................5  أفريقيا الغير عربية.....................................6 | ما هي جنسيتك؟ | .Q8 |
|  | ابتدائي..................................................1  ثانوي....................................................2  جامعى أو أعلى.........................................3  لست متعلما.............................................4 | ما هو أعلى مستوى تعليمي حصلت عليه؟ | .Q9 |
| 2⇨ Q12 | نعم.......................................................1  لا........................................................2 | هل تعمل؟ | .Q10 |
|  | منظمة متعلقه بالصحة.................................1  منظمة غير متعلقه بالصحة............................2  أخرى (حدد)............................................8 | أين تعمل؟ | .Q11 |

|  | الدوحة...................................................1الريان....................................................2  الخور....................................................3  الوكرة...................................................4  الشمال...................................................5  أم صلال................................................6  اداغيان..................................................7 | ما هي منطقة سكنك؟ | .Q12 |
| --- | --- | --- | --- |
|  | أقل من 10000 ريال قطري...........................1  10000- 20000 ريال قطري.......................2  20000 – 30000 ريال قطري......................3  أكثر من 30000 ريال قطري.........................4 | أي من التالي هو أفضل وصف لإجمالي دخل الأسرة الخاص بك؟ | .Q13 |
| \| **استخدام المضادات الحيوية** \| \| --- \| | | | |
| 2⇨ AU5 | نعم......................................................1  لا........................................................2 | هل تم وصف مضاد حيوي لك خلال العام الماضي؟ | .AU1 |
|  | ولا مره.......................................................1  مره............................................................2  مرتين.........................................................3  ثلاث مرات..................................................4  أكثر من ثلاث مرات.......................................5  لا أعلم........................................................6 | كم عدد المرات التي تم وصف مضاد حيوي لك خلال العام الماضي؟ | .AU2 |
| 1 ⇨ AU5 | نعم......................................................1  لا........................................................2  لا اعلم..................................................6 | هل أكملت جرعه المضادات الحيوية الخاصة بك كما تم وصفها؟ | .AU3 |
|  | شفيت قبل نهاية الجرعه...................................1  نسيت أن تأخذ الحبوب.....................................2  كان لها آثار جانبية وأبلغت من قبل الطبيب بوقف تناولها.........................................................3  أبلغت من قبل أحد الأقارب/الأصدقاء بوقف تناولها...4  أخرى (حدد)............................................8 | لماذا لم تكمل الجرعه؟ | .AU4 |
| 2⇨ AU8 | نعم......................................................1  لا........................................................2  لا اعلم..................................................6 | هل استخدمت المضادات الحيوية **بدون ان** يتم وصفها من قبل الطبيب أو طبيب الأسنان خلال العام الماضي (التطبيب الذاتي)؟ | .AU5 |
|  | ولا مره......................................................1  مره...........................................................2  مرتين........................................................3  ثلاث مرات.................................................4  أكثر من ثلاث مرات......................................5  لا أعلم.......................................................6 | كم عدد المرات التي استخدمت من خلالها التطبيب الذاتي بالمضادات الحيوية خلال العام الماضي؟ | .AU6 |
|  | صداع الراس...............................................1  الام الجسم...................................................2  التهاب الحلق................................................3  التهاب الجرح او الجلد....................................4  الحمى........................................................5  البرد والانفلونزا............................................6  إسهال........................................................7  التهاب المسالك البولية....................................8  أخرى (حدد)...........................................8 | ما هي الحاله التي تم استخدام المضادات الحيوية لها بدون وصفة طبية؟ | .AU7 |
|  | نعم.....................................................1  لا.......................................................2  لا اعلم.................................................6 | هل انهيت تناول اخر جرعه من المضادات الحيوية الخاصة بك كما تم وصفها؟ | .AU8 |
|  | نعم.....................................................1  لا.......................................................2  لا اعلم.................................................6 | هل سبق لك استخدام المضادات الحيوية التي كانت وصفت أصلا لعدوى تكررت في وقت لاحق؟ | .AU9 |
|  | نعم.....................................................1  لا.......................................................2  لا اعلم.................................................6 | هل سبق لك استخدام المضادات الحيوية الموصوفة لك في الأصل لنوع آخر من العدوى؟ | AU10 |
|  | نعم.....................................................1  لا.......................................................2  لا اعلم.................................................6 | هل سبق لك الحصول على المضادات الحيوية من صيدليه في الخارج دون وصفة طبية ؟ | .AU11 |
|  | نعم.....................................................1  لا.......................................................2  لا اعلم.................................................6 | هل سبق لك الحصول على المضادات الحيوية من الصيدليات في قطر دون وصفة طبية؟ | .AU12 |
|  | نعم.....................................................1  لا.......................................................2  لا اعلم.................................................6 | هل سبق لك استخدام المضادات الحيوية الموصوفة في الأصل لفرد آخر من العائلة؟ | .AU13 |
|  | نعم.....................................................1  لا.......................................................2  لا اعلم.................................................6 | هل سبق لك استخدام المضادات الحيوية الموصوفة أصلا لشخص آخر لم يكن من أحد أفراد الأسرة؟ | .AU14 |
| \| **هل توافق على العبارات التالية ام لا : استخدام المضادات الحيويه** \| \| --- \| | | | |
|  | لا أوافق بشدة..............................................1  لا اوافق.....................................................2  محايد........................................................3  اوافق........................................................4  اوافق بشده.................................................5 | هناك حاجه لاستعمال مضادات حيويه مختلفه لانواع مختلفه من الامراض | .AG 15 |
|  | لا أوافق بشدة..............................................1  لا اوافق.....................................................2  محايد........................................................3  اوافق........................................................4  اوافق بشده.................................................5 | **المضادات الحيويه فعاله ضد البكتريا** | .AG 1**6** |
|  | للا أوافق بشدة.............................................1  لا اوافق.....................................................2  محايد........................................................3  اوافق........................................................4  اوافق بشده.................................................5 | **يمكن للمضادات الحيويه ان تقتل البكتيريا التي تعيش على الجلد** **وفي القناه الهضميه** | .AG 17 |
|  | لا أوافق بشدة..............................................1  لا اوافق.....................................................2  محايد........................................................3  اوافق........................................................4  اوافق بشده.................................................5 | **المضادات الحيويه تسرع الشفاء من السعال ونزلات البرد** | .AG 1**8** |

|  | لا أوافق بشدة.............................................1  لا اوافق....................................................2  محايد.......................................................3  اوافق.......................................................4  اوافق بشده................................................5 | **المضادات الحيويه تعمل على حالات السعال ونزلات البرد** | .AG 1**9** |
| --- | --- | --- | --- |
|  | لا أوافق بشدة.............................................1  لا اوافق....................................................2  محايد.......................................................3  اوافق.......................................................4  اوافق بشده.................................................5 | **المضادات الحيويه فعاله ضد الفيروسات** | .AG **20** |
|  | لا أوافق بشدة.............................................1  لا اوافق....................................................2  محايد.......................................................3  اوافق.......................................................4  اوافق بشده................................................5 | إ**ذا حصل لديك آثار جانبية أثناء دورة من العلاج بالمضادات** **الحيوية يجب التوقف عن أخذها في أسرع وقت ممكن** | .AG **21** |
|  | لا أوافق بشدة.............................................1  لا اوافق....................................................2  محايد.......................................................3  اوافق.......................................................4  اوافق بشده................................................5 | **إذا كان لديك نوع من ردة الفعل على الجلد عند استخدام المضادات الحيوية، يجب عدم استخدام نفس المضاد الحيوي مرة أخرى** | .AG **22** |
|  | لا أوافق بشدة.............................................1  لا اوافق....................................................2  محايد.......................................................3  اوافق.......................................................4  اوافق بشده................................................5 | **المضادات الحيوية يمكن أن تسبب خلل في توازن البكتيريا المفيدة الموجودة في الجسم** | .AG **23** |
|  | لا أوافق بشدة.............................................1  لا اوافق....................................................2  محايد.......................................................3  اوافق.......................................................4  اوافق بشده................................................5 | **أنا دائما إكمل دورة العلاج بالمضادات الحيوية حتى لو شعرت بتحسن** | .AG **24** |
|  | لا أوافق بشدة.............................................1  لا اوافق....................................................2  محايد.......................................................3  اوافق.......................................................4  اوافق بشده................................................5 | **انه لامر جيد ان تكون قادر على الحصول على المضادات الحيوية من الأقارب أو الأصدقاء دون الحاجة لرؤية الطبيب** | .AG **25** |
|  | لا أوافق بشدة.............................................1  لا اوافق....................................................2  محايد.......................................................3  اوافق.......................................................4  اوافق بشده................................................5 | **أنا أفضل أن اكون قادرعلى شراء المضادات الحيوية من الصيدلية دون وصفة طبية** | .AG **26** |
|  | لا أوافق بشدة.............................................1  لا اوافق....................................................2  محايد.......................................................3  اوافق.......................................................4  اوافق بشده.................................................5 | **انا افضل الاحتفاظ بالمضادات الحيوية في المنزل في حال قد تكون هناك حاجة لها في وقت لاحق** | .AG **27** |

|  | لا أوافق بشدة..............................................1  لا اوافق....................................................2  محايد.......................................................3  اوافق.......................................................4  اوافق بشده................................................5 | **إذا شعرت بتحسن بعد بضعة أيام، أنا احيانا اتوقف عن تناول المضادات الحيوية قبل انتهاء دورة العلاج** | .AG **28** |
| --- | --- | --- | --- |
|  | لا أوافق بشدة.............................................1  لا اوافق....................................................2  محايد.......................................................3  اوافق.......................................................4  اوافق بشده................................................5 | **إذا شعرت بتحسن بعد بضعة أيام، أنا احيانا اتوقف عن تناول المضادات الحيوية قبل انتهاء دورة العلاج** | .AG **29** |
|  | لا أوافق بشدة.............................................1  لا اوافق....................................................2  محايد.......................................................3  اوافق.......................................................4  اوافق بشده................................................5 | **عندما يكون لدي التهاب في الحلق أفضل أن استخدم المضادات الحيوية** | .AG **30** |
| \| **مقدمو الرعاية الصحيه واستخدام المضادات الحيوية** \| \| --- \| | | | |
|  | لا أوافق بشدة.............................................1  لا اوافق....................................................2  محايد.......................................................3  اوافق.......................................................4  اوافق بشده................................................5 | **غالبا ما يشرح الصيادلة لكم كيف ينبغي استخدام المضادات الحيوية** | HC1 |
|  | لا أوافق بشدة.............................................1  لا اوافق....................................................2  محايد.......................................................3  اوافق.......................................................4  اوافق بشده................................................5 | **غالبا يأخذ الأطباء وقتا كافيا لإعلام المريض خلال المعاينة عن كيفية استخدام المضادات الحيوية** | HC2 |
|  | لا أوافق بشدة.............................................1  لا اوافق....................................................2  محايد.......................................................3  اوافق.......................................................4  اوافق بشده................................................5 | **كثيرا ما يصف الأطباء المضادات الحيوية لأن المريض يتوقع ذلك** | HC3 |
|  | لا أوافق بشدة.............................................1  لا اوافق....................................................2  محايد.......................................................3  اوافق.......................................................4  اوافق بشده................................................5 | **غالبا ما يأخذ الأطباء الوقت للنظر بعناية ما إذا كانت هناك حاجة إلى مضادات حيوية أو لا** | HC4 |

شكرا لكم
